# Invariant NKT cells are more abundant in peanut-allergic adults and a subset of CD8^+^ iNKT cells are depleted after peanut oil exposure

**DOI:** 10.3389/fimmu.2023.1293158

**Published:** 2023-11-03

**Authors:** Georgina V. Hopkins, Stella Cochrane, David Onion, Lucy C. Fairclough

**Affiliations:** ^1^ School of Life Sciences, The University of Nottingham, Nottingham, United Kingdom; ^2^ Safety and Environmental Assurance Centre (SEAC), Unilever, Bedford, United Kingdom

**Keywords:** dendritic cells, invariant NKT cells, allergic sensitization, peanut oil, α-galactosylceramide, CD1d, Ara h 8, *in vitro*

## Abstract

**Introduction:**

Peanut allergy is one of the most prevalent food allergies globally. Currently, most research into the mechanisms involved in protein allergy focuses on the protein allergens under investigation, and information on the function of accompanying compounds, such as lipids, is scarce. Thus, this research investigates the role of peanut-associated lipids and invariant natural killer T (iNKT) cells in peanut allergy using a novel, human, *in vitro* assay.

**Methods:**

PBMCs from non-allergic and peanut-allergic subjects were stimulated with the glycolipid, α-Galactosylceramide (α-GalCer), over 14 days for iNKT cell expansion. Autologous dendritic cells (DCs) were stimulated with either peanut oil, the lipid-binding peanut allergen, Ara h 8, or both peanut oil and Ara h 8. The expanded iNKT cells were then immunomagnetically isolated and co-cultured for 5 h with autologous DCs, and cytokine expression was measured by flow cytometry.

**Results:**

A 5-fold higher iNKT cell population was observed in peanut-allergic subject peripheral blood compared to non-allergic controls. In all subjects, conventional flow analysis highlighted iNKTs co-cultured with autologous α-GalCer-pulsed DCs displayed increased IL-4 and IFN-y secretion within 5 hours of co-culture. A 10-parameter unsupervised clustering analysis of iNKT phenotype found significantly more CD3^+^CD8^+^CD25^+^IL-4^+^IL-5^+^IL-10^+^IFNγ^+^ cells in non-allergic adults following culture with peanut oil.

**Conclusion:**

For the first time, we show iNKT cells are more abundant in peanut-allergic adults compared to non-allergic adults, and peanut lipid-exposed iNKT cells resulted in the identification of a subset of CD8^+^ iNKT cells which was significantly lower in peanut-allergic adults. Thus, this study proposes a role for iNKT cells and peanut allergen-associated lipids in peanut allergy.

## Introduction

The prevalence of peanut allergy in Western countries has doubled in the past decade, currently affecting ~1 in 50 individuals (1). Peanut allergy is one of the most prevalent food allergy among children (2, 3), and is a major cause of food allergy-induced fatalities due to the severity of allergic reactions (4). Childhood peanut allergy often continues in adulthood, with approximately 80% of childhood peanut allergy persisting, hence, only 20% outgrow their allergy (5). Furthermore, up to 2.9% of US adults experience peanut allergy, with 17% of these cases developing in adulthood (6). Despite the increasing prevalence in children and adults, and high severity of peanut allergies, the underpinning mechanisms of IgE-mediated food allergy, in particular allergic sensitization (the initial phase of developing an IgE allergy) are still not fully understood.

Importantly, allergen sources, such as peanuts, are not only composed of allergenic proteins, but are accompanied by other compounds, such as lipids. Peanuts contain a high fat content (~50%) and some minor peanut allergens, such as the protein Ara h 8, have been identified as lipid-binding (7). Previous research has shown peanut-derived lipids can interact with peanut allergens to promote a Th2-type allergic response (7, 8). There are several hypothetical mechanisms for the role of allergen source-derived lipids in allergy (9), one of which is the ability of dendritic cells (DCs) to present lipids via CD1d molecules (10) to invariant natural killer T (iNKT) cells and subsequently activate iNKT cells to release Th2 cytokines.

iNKT cells, otherwise known as type I NKT cells, are a subtype of NKT cell, which recognize certain glycolipids by the expression of a TCRβ chain, coupled with an invariant TCRα Vα24-Jα18 chain in humans, and TCRα Vα14-Jα18 in mice (11). Type I iNKT cells can rapidly release immunoregulatory cytokines upon activation by glycolipids. However, very limited iNKT cell numbers are found in humans, comprising approximately 0.01% of the total lymphocytes in healthy human donors (12). In mice, they constitute approximately 1% of lymphocytes (13), thus making them an easy model for iNKT cell study, but the relevance of mice models to human disease is disputed. It is, thus, not surprising that there is limited existing research in this area utilizing human cell-based systems (9).

This paper describes the development and application of a human *in vitro* co-culture system to study the role of peanut lipids and iNKT cells in a model of allergic sensitization. For the first time, iNKT cells from non-allergic and peanut-allergic adults were characterized and co-cultured with DCs pulsed with peanut oil (a source of lipid antigens) with and without addition of the well-established lipid-binding peanut allergen, Ara h 8. Flow cytometric analysis of Th1 and Th2 cytokine production during co-culture was examined to identify any potential influence of these peanut components.

## Materials and methods

### Human subjects

Peanut-allergic subjects were identified using NHS records, with medical notes stating GP-confirmed peanut allergy due to adverse reactions after eating peanuts, including positive relevant test results i.e. RadioAllergoSorbent Testing (RAST) or skin-prick testing. The blood samples were obtained from 11 peanut-allergic adults and 11 non-allergic controls at Cripps Health Centre, Nottingham, by research nurses and transported to the School of Life Sciences, University of Nottingham, for immediate use in experiments (approved by the NHS Health Research Authority Research Ethics Committee (Ref 21/SC/0183)). Information regarding the subject’s age, sex, ethnicity, form of peanut-allergy diagnosis (if applicable), and any other allergies were collected by questionnaire and GP medical records (**Table 1**). Total IgE serum levels were measured by ELISA (ThermoFisher Scientific, UK). A peanut-allergen specific (Ara h2 and Ara h 8) IgE ELISA was developed in-house. Methodology details can be found in ‘Supplementary Methodology’. Total and specific IgE results for all subjects are stated in **Table 1**, and specific IgE results are graphically represented in **Figure S1**.

**Table 1:** Demographics of Subjects.

	Non-Allergic	Peanut-Allergic
**Number of Participants**	11	11
**Mean Age (Range)**	30.5 years (20-54)	23.9 years (21-32)
**Sex**	Female: 6 Male: 5	Female: 6 Male: 5
**Ethnicity**	White British: 8 White Polish: 1 Latino: 1 Asian: 1	White British: 10 Mixed Race: 1
**Peanut Allergy Diagnosis**	N/A	GP-confirmed: 11 Positive RAST and/or positive Skin-prick test: 11
**Other IgE Allergies**	N/A	Cats: 7 Birch Pollen: 6 Grass Pollen: 6 Other nuts: 4 Dust mites: 2 Dogs: 2 Mould: 2 Apples: 2 Peaches: 2 Cherries: 2 Sesame: 1 Cucumbers: 1 Courgettes: 1 Pears: 1 Nectarines: 1 Plums: 1 Kiwi: 1
**Mean Total IgE (Range)**	50.77 ng/mL (7.4 - 151.3)	253.6 ng/mL (42.8 – 957.2)
**Mean Peanut-specific IgE (Range)**	Ara h 2: 0.107 O.D. (0.062 – 0.248) Ara h 8: 0.103 O.D. (0.072 – 0.147)	Ara h 2: 0.365 O.D. (0.099 – 0.842) Ara h 8: 0.168 O.D. (0.090 – 0.530)

The number of subjects, their age, sex, ethnicity, form of peanut allergy diagnosis, any other allergies, total serum IgE levels, and Ara h 2 and Ara h 8 specific IgE levels, for non-allergic and peanut-allergic subjects. N/A, not applicable.

### iNKT cell expansion

Peripheral blood mononuclear cells (PBMCs) were isolated from 50 mL of human blood samples using SepMate™-50 Tubes (StemCell Technologies, UK) for density gradient centrifugation. Isolated PBMCs were cultured at 1x10^6^ cells/mL in RPMI (Merck, UK) supplemented with 10% human AB serum (Merck, UK), in addition to 50 U/mL IL-2 (Miltenyi Biotec, UK) and 100 ng/mL α-Galactosylceramide (α-GalCer) (Abcam plc., UK). Dimethyl sulfoxide (DMSO) was used as a control, as α-GalCer was re-constituted in DMSO. The cells were incubated at 37°C for up to 14 days, supplementing with IL-2 every 4 days. Some iNKT cells were stimulated with 1 mg/mL peanut oil (Handa Fine Chemicals, UK) for 14 days to examine any expansion.

### iNKT cell immunomagnetic isolation

Expanded iNKT cells were stained with the α-GalCer-loaded CD1d Tetramer R-Phycoerythrin (R-PE). Ultrapure anti-PE Microbeads (Miltenyi Biotec, UK) were mixed into the cell solution to bind to any Tetramer PE-labelled iNKT cells and finally, the iNKT cells were positively selected by applying the cell solution to a MACS MS column. iNKT cell purity was analyzed by flow cytometry, staining for CD3 and α-GalCer-loaded CD1d Tetramer R-PE.

### Lipid-pulsed DCs

At Day 8 of iNKT cell expansion, 6 peanut-allergic and 6 non-allergic subjects returned for a second blood donation and autologous PBMCs were isolated. Demographics and IgE results for these subjects are provided in **Table S1**.

The PBMCs were mixed with anti-CD14 Microbeads (Miltenyi Biotec, UK) and applied to a MACS LS column for positive selection of CD14^+^ monocytes. The monocytes were then cultured at 5 x10^5^ cells/mL with 50 ng/mL GM-CSF and 20 ng/mL IL-4 (Miltenyi Biotec, UK) to generate immature DCs (iDCs). The monocytes were cultured for 5 days, replenishing media and cytokines on Day 3.

Once iDCs were generated, the iDCs were then stimulated for 24 hours with either 100 ng/mL α-GalCer, 0.1% DMSO control, 1 mg/mL Peanut Oil (Handa Fine Chemicals, UK), 10 µg/mL Ara h 8, or both peanut oil and Ara h 8. The α-GalCer-pulsed DCs were used as a positive control, as it is well-characterized for activating iNKT cells to produce cytokines upon DC-presentation to the iNKT cell. Prior to iDC exposure, the peanut oil was sonicated for 1 hour at 25 kHz in 0.1% DMSO to help solubilize the lipid. For Peanut oil specification see **Figure S2**.

### Co-culture of DCs and iNKT cells

Expanded iNKT cells were co-cultured with autologous DCs at a ratio of 1:2 (DC:iNKTs). The cells were cultured in RPMI supplemented with 10% human AB serum, and 1X protein transport inhibitor cocktail (ThermoFisher Scientific, UK) was added for 5 h at 37°C to block cytokine release from the cells, enabling intracellular staining of the cytokines.

### Flow cytometry

Cells were removed from culture and centrifuged with 1 mL of phosphate buffer albumin (PBA) at 300g, for 5 minutes before aspirating the supernatant and re-suspending the pellet. All cells were first stained with either 2 µg/mL of anti-CD1d-GalCer Tetramer (Proimmune, Oxford, UK) or 2 µg/mL of a blank-loaded tetramer (Proimmune, Oxford, UK) which was used as a negative control, for 30 minutes at 4°C. The following antibodies were then added to the cells and incubated at 4°C for a further 30 minutes: 1 µg/mL Zombie NIR dye (Biolegend, UK) to identify and exclude dead cells from the analysis, 3 µg/mL anti-CD3 (Biolegend, UK), 8 µg/mL anti-CD4 (BDbiosciences, UK), and 4 µg/mL anti-CD8 (Miltenyi, UK) to phenotype tetramer positive iNKT cells, 8 µg/mL anti-CD209 (Biolegend, UK) was used to identify DCs, and finally, 8 µg/mL of anti-CD19 (Biolegend, UK) was used to remove B cells from analysis, as B cells can also bind to the CD1d-GalCer Tetramer. After antibody incubation, PBA was subsequently added and the cells were centrifuged for 5 minutes at 300g. The supernatant is then completely removed and cell pellet is re-suspended in 200µL of fixation buffer (4% formaldehyde fix in 1X PBS) overnight at 4°C. The fixation buffer was then washed off by adding PBA and centrifuging for 5 minutes at 300g. The cell pellet was re-suspended in permeabilization buffer (ThermoFisher Scientific, UK) and centrifuged for 5 minutes at 300g. Supernatant was aspirated and 4 µg/mL of IL-4 and IFN-y, 8 µg/mL of IL-10 (Biolegend, UK), 2 µg/mL of IL-5 and 1 µg/mL of IL-12 (Miltenyi Biotec, UK) were added for 30 minutes at room temperature. Finally, the cells were centrifuged for 5 minutes at 300g, before aspirating the supernatant completely and tapping to re-suspend. The cells were then re-suspended in fixation buffer and analyzed using ID7000 Spectral Cell Analyzer (Sony, UK). The FCS data were then analyzed using Kaluza software.

### Clustering analyses

Multi-dimensional clustering analysis was performed on iNKT cells co-cultured with peanut oil-stimulated DCs and α-GalCer-stimulated DCs, analyzing with parameters CD1d-αGC tetramer, CD3, CD4, CD8, CD25, CD69, IFNγ, IL-4, IL-5, and IL-10, using FlowJo v10 (Beckman Dickinson) and X R plugins. Single viable CD3/CD1d-αGalCer Tetramer iNKT cells were first gated in FlowJo before equal sampling of 5,000 events from each of the 6 non-allergic and 6 peanut-allergic subjects. FlowSOM clustering was performed to obtain meta-clusters (14) which could then be presented on a t-distributed stochastic-neighbor embedding (tSNE) plot. This was performed with opt-SNE learning configuration, 1000 iterations, a perplexity of 30, learning rate of 3500, Exact (vantage point tree) KNN and Barnes-Hut gradient algorithm (15).

### Statistical analyses

The comparison of iNKT cell numbers between groups, before expansion, was statistically analyzed using an unpaired t-test. Statistical testing on the multi-dimensional clustering analysis was a two-way ANOVA with Sidak’s multiple comparisons. All other data were analyzed by mixed effects analyses with Sidak’s multiple comparison. All statistical analyses were performed using GraphPad Prism 9.3.1. p-values <0.05 were considered significant for experiments.

### Method overview

**Figure 1:**
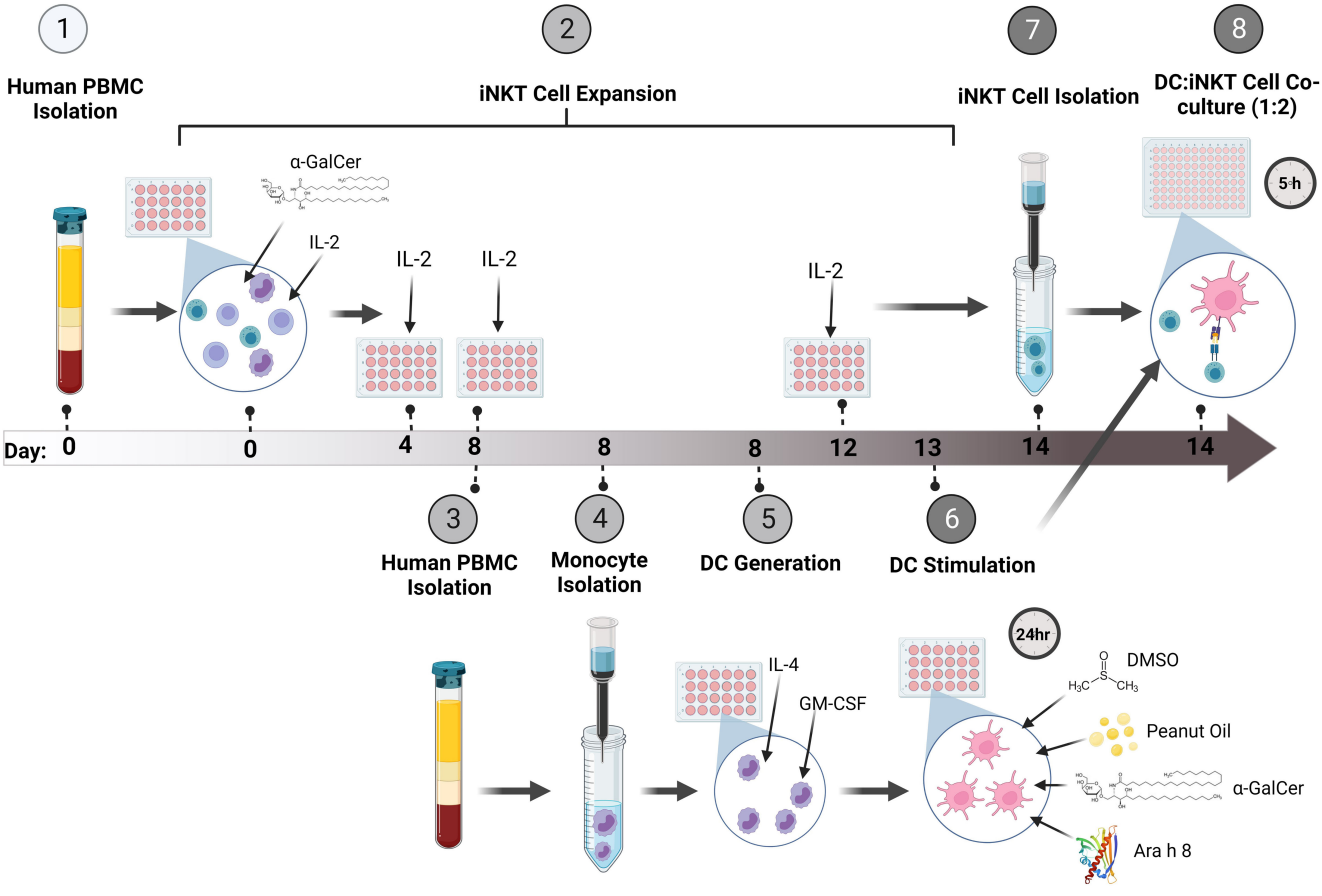
Method Overview. (1) Firstly, PBMCs were isolated from whole human blood and (2) stimulated with the glycolipid, α-GalCer, and IL-2 to induce iNKT cell expansion. The cells were incubated for up to 14 days, re-stimulating the culture with IL-2 every 4 days. (3) At Day 8 of iNKT expansion, blood was obtained from the same donor and PBMCs were isolated. (4) Monocytes were isolated using CD14+ immunomagnetic isolation. (5) The monocytes were incubated with GM-CSF and IL-4 for 5 days to generate immature DCs (iDCs). (6) Once iDCs were generated, the DCs were pulsed with either α-GalCer, DMSO, Peanut Oil, Ara h 8, or both Peanut Oil and Ara h 8, and incubated for 24 hours to allow DC uptake of the lipids and/or allergens. (7) At Day 14 of iNKT cell expansion, Tetramer positive iNKT cells were immunomagnetically isolated. The iNKT cells could then be isolated by using anti-PE immunomagnetic selection. (8) The isolated iNKT cells and the lipid/allergen-pulsed DCs were then co-cultured together at a ratio of 1:2 (DC:iNKT) for 5 hours. Th1 and Th2 cytokine production was then measured by flow cytometry. Created using BioRender.

An overview of the system for expanding iNKT cells and co-culturing them with peanut lipid and/or allergen-pulsed DCs is presented in **Figure 1**.

## Results

### Method development

**Figure 2:**
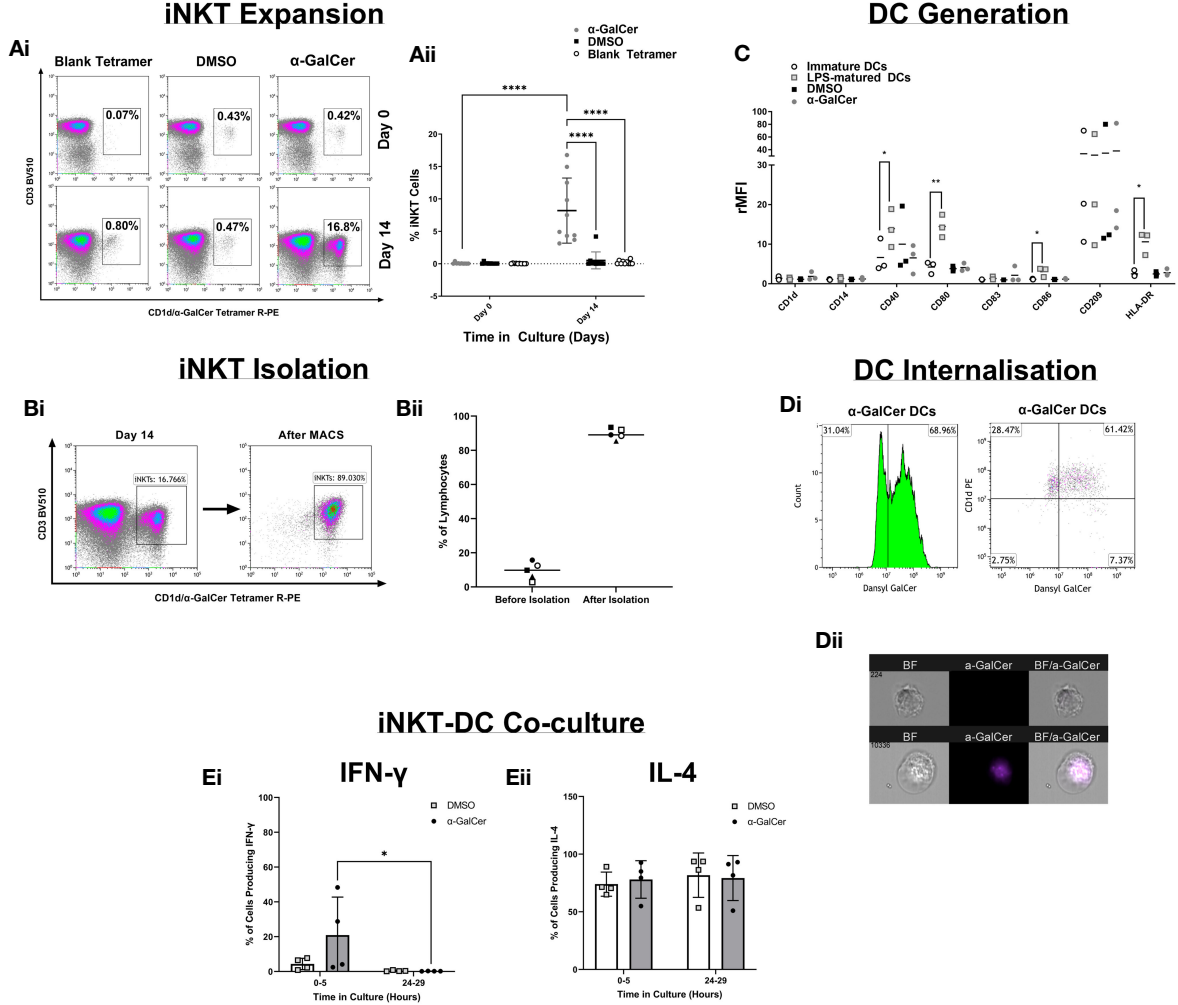
Method Development. **(Ai)** Density plots demonstrating the percentage of CD3+/CD1d-aGC Tetramer+ iNKT cells after PBMC stimulation with α-GalCer or the DMSO control, or after staining with a blank CD1d tetramer. **(Aii)** iNKT cell expansion across 9 healthy donors. **(Bi)** Density plots of iNKT cells before iNKT cell immunomagnetic isolation from PBMCs, and after iNKT isolation. **(Bii)** iNKT cell purity after isolation, across 4 healthy donors. **(C)** DC marker analysis of Immature DCs generated from human CD14+ monocytes and stimulation with LPS, α-GalCer, or the DMSO control. **(Di)** The percentage gated of immature DCs positive for the expression of dansylated α-GalCer and CD1d, after 24 hours of stimulation. **(Dii)** Immature DC Internalization of dansylated α-GalCer (purple) by imaging cytometry. **(Ei)** A comparison of 0-5 hours and 24-29 hours of iNKT-DC co-culture length to detect IFNγ cytokine production and **(Eii)** IL-4 production (n=4). *p<0.05, **p<0.01, ****p<0.0001.

To develop a robust and reproducible assay many components of the method were optimized (**Figures 2A–D**). iNKT cell expansion was first optimized with the glycolipid, α-GalCer (**Figure 2A**), showing iNKT cells could be expanded. iNKT cells were then isolated and cell purity was tested by flow cytometry (**Figure 2B**), reproducibly giving above 80% purity. DCs were generated from monocytes and immature DC (iDC) marker expression was compared to iDCs stimulated with lipopolysaccharide (LPS), DMSO, or α-GalCer (**Figure 2C**), showing upregulation of the key markers CD40, CD80, CD86, and HLA-DR following LPS activation. To show lipid could be internalized, the iDC surface and intracellular expression of a fluorescent derivative of α-GalCer, dansylated α-GalCer was tested by flow cytometry, showing internalization after 24 hours of incubation with dansylated α-GalCer, with an internalization score above 0 (score=2.40) (**Figure 2D**). Finally, the timing of iNKT : DC co-culture was tested (**Figure 2E**), highlighting cytokine changes predominately occurred 0-5 hours after co-culture. Further detail of these optimization experiments are presented in ‘Supplementary Text 1’.

### Higher abundance of iNKT cells in peanut-allergic adults

**Figure 3:**
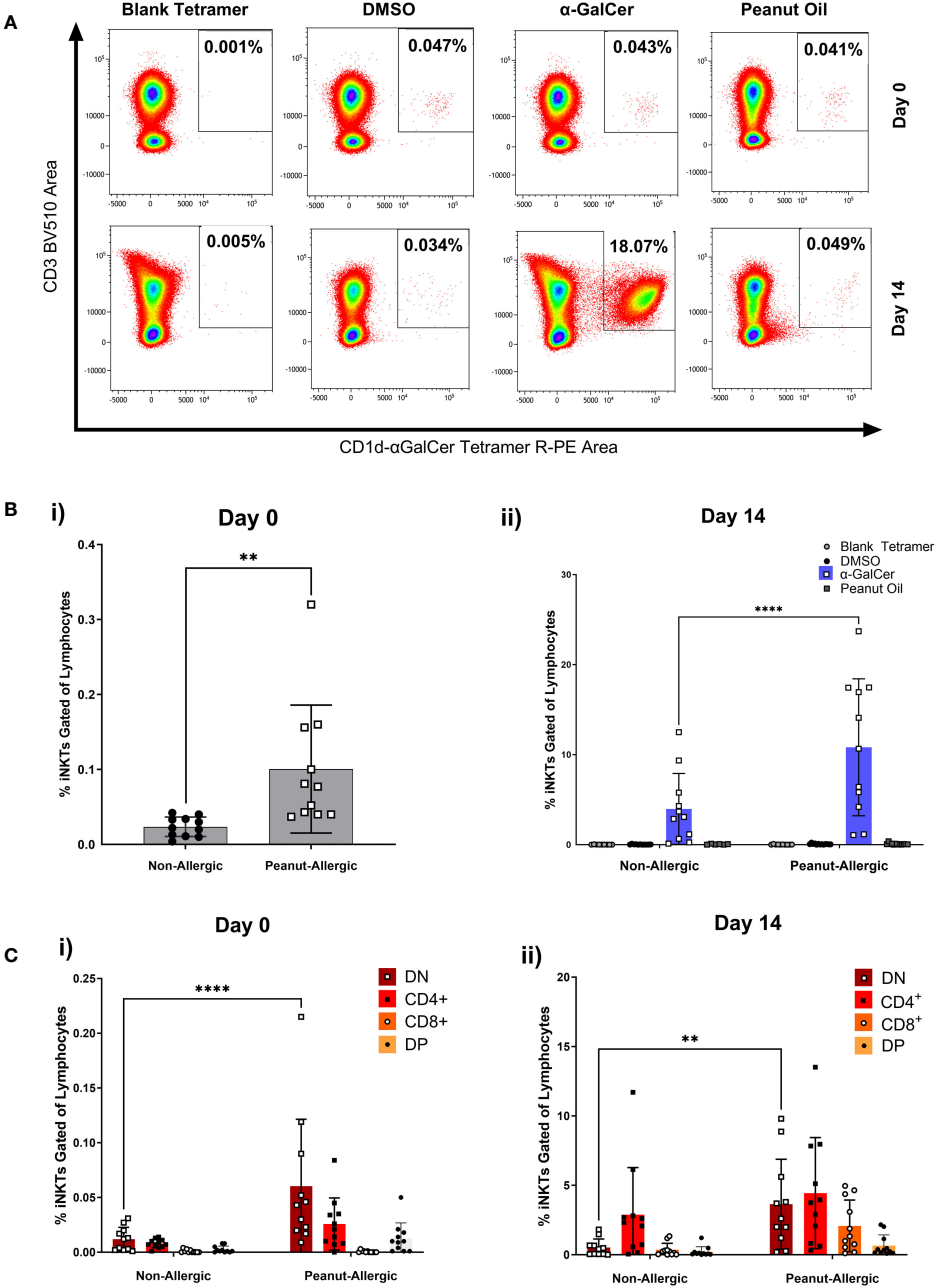
Peripheral iNKT Cell Abundance and Expansion in Non-allergic and Peanut-Allergic subjects. **(A)** Representative density plots of a peanut allergic subject’s iNKT cell expansion. **(Bi)** Percentage of iNKT cells gated on CD19^-^ lymphocytes before expansion, analyzed by an unpaired t-test, and **(Bii)** after 14 days of expansion with DMSO, α-GalCer, peanut oil, or stained with the negative tetramer control. **(Ci)** Non-allergic and peanut-allergic iNKT cell subsets before expansion (Day 0). **(Cii)** Non-allergic and peanut-allergic iNKT cell subsets after expansion (Day 14). Data in Bi was analyzed by an unpaired t-test, and data in Bii-Cii was analyzed by a mixed effects analysis with Sidak’s multiple comparisons. (n= 11 non-allergic and 11 peanut-allergic subjects, **p<0.01, ****p<0.0001).

iNKT cell populations were defined by the expression of CD3 and α-GalCer-loaded CD1d tetramer. **Figure 3A** demonstrates this gating using a peanut-allergic example, which highlights the number of iNKT cells before and after 14 days of exposure to α-GalCer, peanut oil, the DMSO control, or using a blank-loaded tetramer to identify any non-specific binding of the CD1d-αGalCer tetramer. Before iNKT cell expansion, peanut-allergic subjects had a significantly larger population of iNKT cells, with a mean of 0.10% compared to non-allergic subjects, with a mean of 0.02% iNKT cells (p<0.01) (**Figure 3Bi**). After 14 days of stimulation with α-GalCer and IL-2, the percentage of iNKT cells was again significantly higher in the peanut-allergic subjects compared to the non-allergic subjects (p<0.0001) (**Figure 3Bii**). The DMSO, blank tetramer control, and peanut oil caused no iNKT cell expansion. Flow cytometric analysis of iNKT cells phenotype at day 0 (before expansion) showed peanut-allergic subjects to have a significantly higher number of double negative (CD4^-^CD8^-^) iNKT cells, compared to non-allergic controls (p<0.0001) (**Figure 3Ci**). After 14 days of iNKT cell expansion, DN (double negative) iNKT cells were still significantly higher in peanut-allergic subjects (p<0.01) (**Figure 3Cii**). Moreover, a small DP (double positive) iNKT subset was identified, which did not differ between subject groups. Also, as the mean age of non-allergic and peanut-allergic subjects were different, an unpaired t-test was conducted which demonstrated no significant differences in age between groups (p>0.05) (data not shown), confirming these iNKT cell differences were not due to differences in the group ages. Overall, iNKT cells from peanut-allergic individuals are in higher abundance before and after iNKT cell expansion, with a high DN subset before expansion, and increased CD4^+^ iNKT cells after expansion.

### Increased IL-4 and IFN-γ cytokine production by iNKT cells after α-GalCer exposure

**Figure 4:**
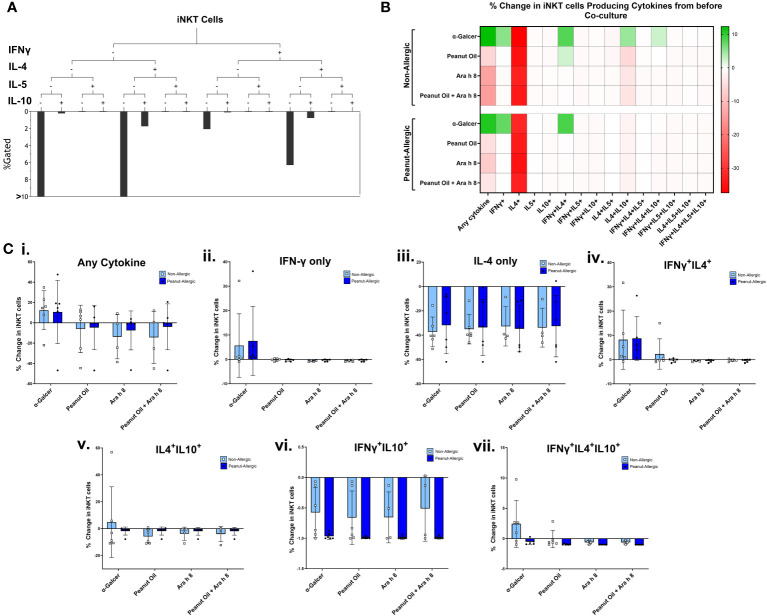
iNKT Cell Cytokine Production during Co-culture with DCs. **(A)** A representative tree diagram to depict the Boolean gating which was performed for iNKT cell cytokines IFN-γ, IL-4, IL-5, and IL-10, producing percentages for each combination of cytokines expressed by the iNKT cells. **(B)** The percentage of iNKT cells producing these different combinations of cytokines are presented as a heat map, with green indicating an increase, and red indicating a decrease. Non-allergic (top) and peanut-allergic (bottom) iNKT cells after co-culture with either, α-GalCer-pulsed DCs, peanut oil-pulsed DCs, Ara h 8-pulsed DCs, or peanut oil + Ara h 8-pulsed DCs are displayed (n=6 non-allergic, 6 peanut-allergic). The percentage of iNKTs producing the cytokines is relative to before co-culture and minus the DMSO control (background). Calculated by subtracting the DMSO background and then dividing the % of iNKT cells producing the cytokines in each condition, by the % of iNKT cells producing the cytokines before co-culture. **(C)** Graphical representation of heatmap data; the percentage change in iNKT cell cytokine production relative to before co-culture with DCs for **(i)** any cytokine, **(ii)** IFN-γ only, **(iii)** IL-4 only, **(iv)** IFNγ^+^IL4^+^, **(v)** IL4^+^IL10^+^, **(vi)** IFNγ^+^IL10^+^, **(vii)** IFNγ^+^IL4^+^IL10^+^. Differences in cytokine production was statistically analyzed by a mixed effects analysis with Sidak’s multiple comparisons.

A subset of 6 subjects in each group returned for a second blood donation, allowing autologous DCs to be generated. Expanded iNKT cells were then co-cultured with peanut lipid and/or Ara h 8-pulsed DCs, to allow DCs to present the lipid to iNKT cells. iNKT co-cultures with α-GalCer and DMSO-pulsed DCs were used as positive and negative controls, respectively. Cells were examined by flow cytometry profiling iNKT phenotype markers (CD1d-αGalCer Tetramer and CD3), and intracellular cytokines (IFNγ, IL-4, IL-5, and IL-10). iNKT cell gating strategy and cytokine fluorescence minus one (FMO) controls can be found in **Figure S3**. Boolean gating, performed using gates from bivariate FACS plots (a representative sample is shown in **Figure S4**), allowed combinations of IFNγ, IL-4, IL-5, and IL-10-producing iNKT cells to be measured, as shown in **Figure 4A**. A heat map of the percentage of iNKT cells producing these cytokine combinations at 0-5 hours of co-culture with DCs is shown in **Figure 4B**, across all subjects, with individual data points shown in **Figure 4C**. The data illustrates the percentage of iNKT cells producing cytokines, relative to before being placed in culture with DCs (i.e. relative to day 14 of iNKT cell expansion) and minus the DMSO background.

The data highlights both subject groups exhibit an increased iNKT total cytokine response to the positive lipid control, α-GalCer, whereas reduced cytokine responses were observed in the peanut oil and/or Ara h 8 co-cultures, compared to Day 14 cytokine production. The increased total response by α-GalCer exposure was largely due to an increase in the percentage of iNKT cells producing IFN-γ. Furthermore, iNKT cells producing only IL-4 decreased across all conditions, in both subject groups, but there were notable increases in the percentage of iNKT cells producing both IFNγ+IL-4+ in both subject groups, in response to α-GalCer only. Finally, increased IL-4+IL-10+ iNKT cells and IFNγ+IL-4+IL-10+ iNKT were observed in non-allergic iNKT cells responding to α-GalCer.

### A cluster of CD8^+^ iNKT cells identified in response to peanut lipids in non-allergic adults

**Figure 5:**
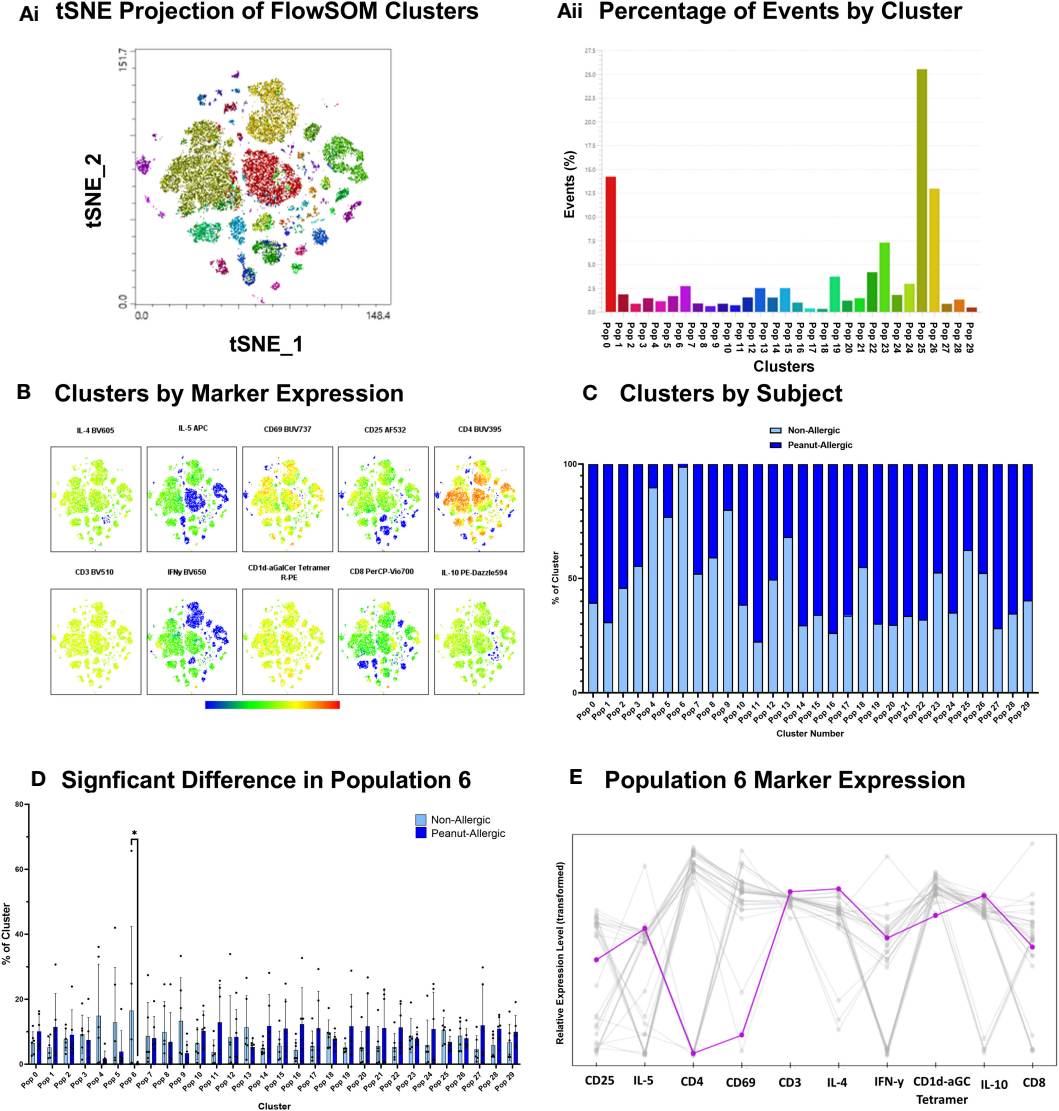
Peanut Oil-exposed iNKT Cell Clustering Analysis. Peanut Oil iNKT Cell Clustering Analysis. **(Ai)** FlowSOM clusters from peanut oil-exposed iNKT cells presented as a tSNE plot. **(Aii)** FlowSOM clusters by % of events. **(B)** Heatmap of Clusters by marker expression. **(C)** % of cluster occupied by non-allergic or peanut-allergic iNKT cells. **(D)** A two-way ANOVA with Sidak’s multiple comparisons was performed to test statistical differences between non-allergic and peanut-allergic subjects, within each cluster. **(E)** Marker expression of ‘population 6’. n= 6 non-allergic, 6 peanut-allergic, *p<0.05.

Clustering analysis was performed using FlowSOM algorithm for an unsupervised analysis of iNKT cells exposed to peanut oil-pulsed DCs. Cells were profiled for iNKT phenotype markers (CD1d-αGalCer Tetramer, CD3, CD4, and CD8), activation markers (CD25 and CD69), and intracellular cytokines (IFN-γ, IL-4, IL-5, and IL-10). 30 clusters were identified (**Figures 5Ai-Aii**) and their marker expression presented in **Figure 5B**. The percentage of iNKT cells belonging to peanut-allergic or non-allergic subjects within each cluster was determined (**Figure 5C**) and statistical analysis found ‘population 6’ was significantly higher in non-allergic compared to peanut-allergic subjects (p<0.05) (**Figure 5D**). Cluster Explorer analysis was performed which found ‘population 6’ was CD8^+^ iNKT cells with late activation that are producing IFN-γ, IL-4, IL-5, and IL-10 (**Figure 5E**). Multi-dimensional clustering analysis was also performed for the positive control, α-GalCer, which found no significant differences between subject groups (**Figure S5**).

### Reduced DC IL-10 production following α-GalCer stimulation in peanut-allergic adults

**Figure 6:**
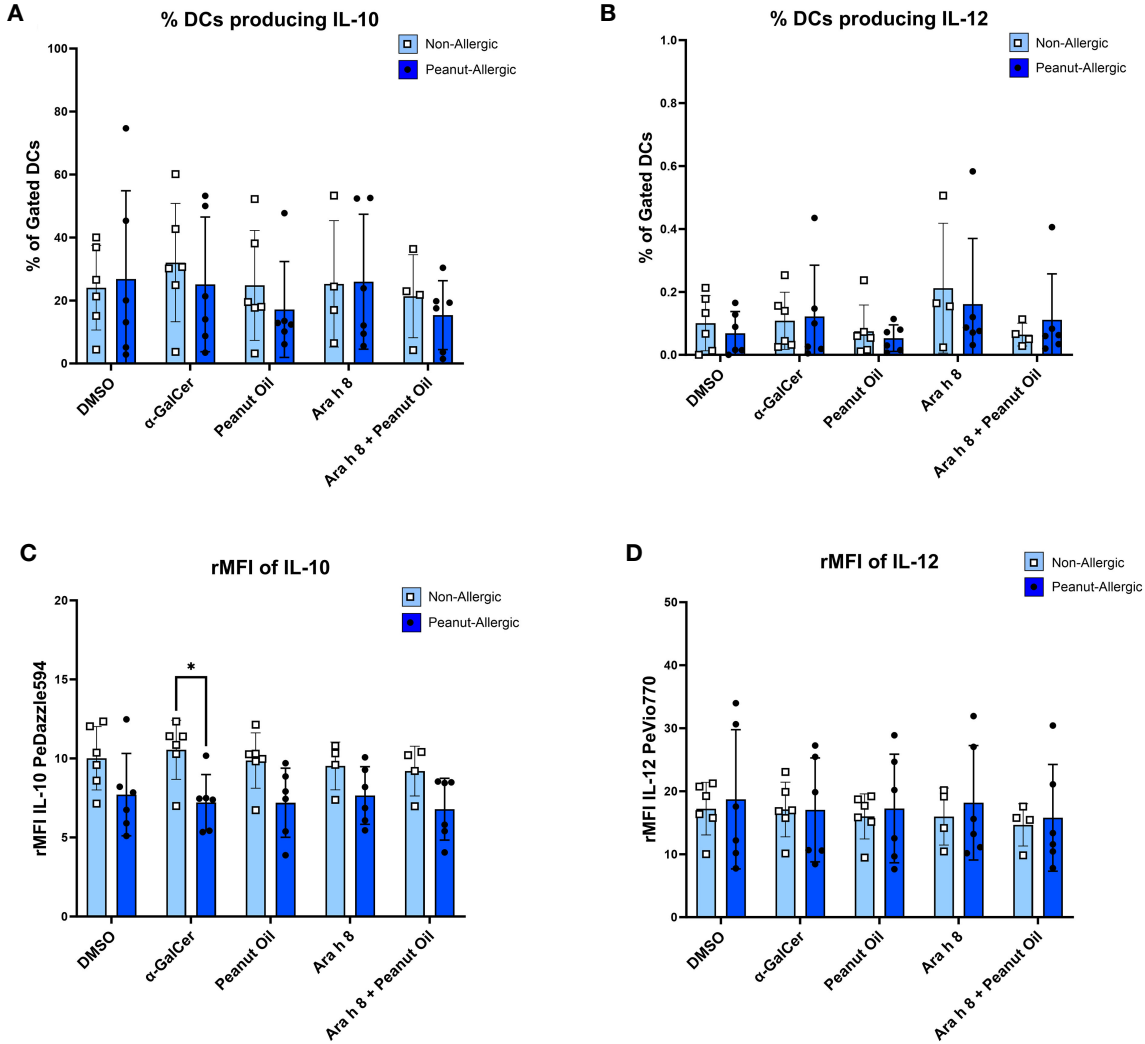
DC Cytokine Production during Co-culture. The cytokine production from DCs stimulated with either the DMSO control, α-GalCer, peanut oil, Ara h 8, or both Ara h 8 and peanut oil, after 5 hours of co-culture with autologous iNKT cells. Cytokines were stained for and analyzed by flow cytometry. The graphs represent **(A)** % DCs producing IL-10 **(B)**, rMFI of IL-10, **(C)** % DCs producing IL-12, **(D)** rMFI of IL-12. All results are comparing DCs from non-allergic and peanut-allergic subjects. A mixed-effects analysis with Sidak’s multiple comparisons was conducted. (*p<0.05, vertical bars represent standard deviation, n=6 non-allergic, 6 peanut-allergic).

DCs were gated for (**Figure S6**) and DC cytokine production during the iNKT-DC co-culture was then also assessed by flow cytometry. Representative FACS plots are shown in **Figure S7**. Cells were flow cytometry profiled with DC marker CD209 and cytokines IL-10 and IL-12. Peanut-allergic DCs stimulated with α-GalCer exhibited significantly higher IL-10 production (Mean rMFI= 10.54, p<0.05) than non-allergic subjects (Mean rMFI= 7.21) (**Figure 6C**). No other significant differences in terms of rMFI or the % of DCs producing IL-10 or IL-12 between any condition or subject group were noted (**Figures 6A, B, D**).

## Discussion

Due to the extremely low abundance of iNKT cells in human peripheral blood, research into the role of human iNKT cells in allergy is challenging. The existing, but limited, research into lipids in allergy mainly utilize murine models (9). This study developed a human, *in vitro* co-culture system for investigating the role of lipids, iNKT cells, and DCs in allergy. Thus, this is the first known study to demonstrate significantly higher iNKT cells in adults with peanut allergy, indicating a role in allergic disease.

Peanut allergy is one of the most prevalent childhood allergies, but unlike with childhood allergies to milk and egg, there is a failure to gain tolerance in the majority of cases, with approximately 80% of peanut-allergic children remaining allergic in adulthood (16). Thus, this study recruited peanut-allergic adults to investigate the role of peanut lipids in peanut allergy. Different exposure routes can lead to peanut allergy, with primary peanut allergy occurring from sensitization to peanut only and peanut allergy due to cross reactivity with tree or grass pollen (secondary allergy), which is typically associated with milder oral allergy symptoms. To establish if a person is peanut allergic and distinguish if they have primary or secondary peanut allergy, it can be advantageous to measure IgE to multiple peanut components (17). Access to a multiplex diagnostic array was not available for this study, but subjects’ levels of IgE against both Ara h 2 and Ara h 8 were measured. Ara h 2 was chosen as a target as it is a major allergen utilized for diagnosing peanut allergy (18) and IgE to Ara h 8 was measured as this was the lipid binding peanut allergen used in the study. The results show that the peanut-allergic subjects had a significantly higher mean Ara h 2-IgE compared to non-allergic subjects. Low Ara h 2-specific IgE results from some peanut allergic subjects indicates these individuals were sensitized to other peanut allergens, as indicated by some high Ara h 8 IgE results, in addition to all allergic subjects being GP-confirmed peanut allergic, with a positive RAST and/or skin prick test result at the time of diagnosis.

Like all immune cells, iNKT cells circulate the blood before recruitment into tissues. But there is currently much debate about levels of iNKT cells in allergic disease, with evidence showing fewer peripheral blood iNKT cells in milk-allergic children (19), but increased iNKT cells in children with allergic asthma (20). There is also data which suggests NKT cell numbers are not altered in allergic disease (21), although this study measured CD3^+^CD56^+^ NKT cells, not invariant NKT cells. Here, we focused on adults over 18 years of age with allergy which identifies those individuals who do not outgrow their childhood peanut allergy. Thus, this is the first evidence of differences in iNKT cell populations in adults and specifically in peanut-allergy. We demonstrate a 5-fold higher population of iNKT cells in peanut-allergic adults compared to non-allergic adults, from peripheral blood lymphocytes. This increased iNKT cell population may be specific to this allergen, where proteolytically active proteins may drive sensitization through a mechanism involving iNKT cells. Furthermore, CD4/CD8 phenotyping of the iNKT cells revealed DN iNKT cells are significantly higher in peanut-allergic compared to non-allergic adults. After expansion with α-GalCer, iNKT cells from peanut-allergic adults expanded more readily, likely due to the higher initial iNKT cell population, and shifted to a CD4^+^ iNKT phenotype. This shift in phenotype after expansion with α-GalCer is consistent with previous literature (22). Furthermore, in agreement with some studies (23), we show that there is a small (~0.001% of lymphocytes) CD4^+^CD8^+^ (DP) iNKT cell population, but this contrasts to some studies which suggest there are no iNKT cells that are DP (22, 24). Identifying these different iNKT cell subsets is important as they have different cytokine profiles, with CD4^+^ iNKT cells predominantly producing IL-4, and DN or CD8^+^ iNKT cells predominantly producing IFN-γ (16).

In addition to α-GalCer, PBMCs were also stimulated with peanut oil however, this caused no iNKT cell expansion after 14 days. This is in contrast to another study that stimulated iNKTs from children with food allergy with the lipid milk sphingomyelin, and found the iNKTs proliferated in response (25). This might have occurred here because a highly refined peanut oil was utilized rather than a specific lipid class, resulting in lower abundance of the activating lipid. It is also possible that the refinement process could have impacted upon the lipid composition of the peanut oil (26).

DCs are critical for the presentation of lipids to iNKT cells, via CD1d molecules (27). iNKT cells recognize the lipid via an invariant T cell receptor (iTCR) which activates the iNKT cell to secrete Th1 and/or Th2 cytokines. The DC:iNKT co-culture utilized here indicates there were increases in IFN-γ^+^ and IFN-γ^+^IL4^+^ producing iNKT cells but only in response to the positive control, α-GalCer pulsed DCs, in both non-allergic and peanut-allergic subjects. Although, a murine study of NKT cells found IL-4 production during sensitization was not dependent on NKT cells (28). Furthermore, peanut oil exposure with or without the lipophilic peanut allergen, Ara h 8, did not influence cytokine production. Although, clustering analysis of iNKT cells co-cultured with peanut oil-pulsed DCs found one iNKT cell type was more abundant in non-allergic subjects, which was CD8^+^ iNKT cells with late activation that are producing IFN-y, IL-4, IL-5, and IL-10 cytokines. CD8^+^ iNKT cells primarily produce IFN-γ (22), but this indicates there are small CD8^+^ populations which can also produce Th2 cytokines. This highlights the importance of unbiased, multi-dimensional flow analysis to identify cell populations which would not have been recognized during conventional flow analysis. Clustering analysis of iNKT cells co-cultured with the positive control, α-GalCer-pulsed DCs, showed no differences between subject groups, highlighting this small CD8^+^ iNKT cell population is specific to peanut-oil exposure.

DC cytokine production was also analyzed as this may influence subsequent cytokine production by iNKT cells. Interestingly, non-allergic α-GalCer-stimulated DCs produced a significantly higher amount of IL-10 than peanut-allergic DCs. This is supported by another study which found human moDCs treated with IL-10 suppressed allergen-induced T-cell proliferation of CD4^+^ T helper cells and Th2 cytokine release, promoting tolerance of the allergen (29). Thus, the production of IL-10 suggests the non-allergic DCs may be promoting tolerance, as IL-10 is a powerful anti-inflammatory cytokine which can induce Tregs with suppressive functions (30). Also, in this study there were no differences in IL-12 production by moDCs between non-allergic and allergic individuals, which is expected (31).

## Conclusion

A human *in vitro* co-culture system to examine the role of lipids in IgE-mediated allergy was developed. Peanut-allergic individuals exhibit higher iNKT cells than non-allergic and produce IL-4 and IFN-γ when co-cultured with α-GalCer pulsed DCs. Furthermore, iNKT cells exposed to DCs loaded with peanut lipids and/or Ara h 8 did not alter iNKT cell cytokine production. A significantly smaller CD8^+^ iNKT cell population producing IFN-γ, IL-4, IL-5, and IL-10 in peanut-allergic adults was identified after exposure to DCs loaded with peanut oil, suggesting that despite a higher overall iNKT cell population, certain iNKT cell responses may be reduced in allergy. Lastly, in non-allergic donors, dendritic cells produced higher levels of the regulatory cytokine, IL-10. Thus, this data provides evidence for an association between iNKT cells and allergic disease.

## Data availability statement

The original contributions presented in the study are included in the article/**Supplementary Material**. Further inquiries can be directed to the corresponding author.

## Ethics statement

The studies involving humans were approved by NHS Health Research Authority Research Ethics Committee. The studies were conducted in accordance with the local legislation and institutional requirements. The participants provided their written informed consent to participate in this study.

## Author contributions

GH: Formal Analysis, Investigation, Methodology, Writing – original draft, Writing – review & editing. SC: Conceptualization, Funding acquisition, Supervision, Writing – review & editing. DO: Conceptualization, Funding acquisition, Supervision, Writing – review & editing. LF: Conceptualization, Funding acquisition, Project administration, Resources, Supervision, Writing – review & editing.
