# Supplementary material for: Invariant NKT cells are more abundant in peanut-allergic adults and a subset of CD8+ iNKT cells are depleted after peanut oil exposure
Source: Front Immunol. 2023 Nov 3;14:1293158. doi: 10.3389/fimmu.2023.1293158 (PMC10655100; doi:10.3389/fimmu.2023.1293158)

**Supplementary Methodology**

**Total IgE ELISA**

Briefly, wells of a 96-well plate were coated with 100 µL of capture anti-human IgE, diluted 1:250 in coating buffer (1X PBS). The plate was sealed with aluminium tape and incubated at 4°C overnight. The plates were then washed twice with wash buffer (PBS-0.05% Tween-20), soaking for 1 minute between washes, and blocked with 250 µL of blocking buffer (PBS with 1% Tween-20 and 10% BSA) overnight at 4°C. After incubation, the blocking buffer was aspirated and the plate washed twice. Then, 100 µL of serially diluted human IgE standard was added to each well in duplicate, to produce a standard curve. Plasma samples were stored at 1:2 dilution in PBS, so samples were thawed and diluted 1:5 in Assay buffer (PBS with 1% Tween™ 20, 10% BSA) to result in a final 1:10 dilution. 100 µL of diluted sample was added to each well in duplicate, and assay buffer alone was used as blank. The plate was sealed and incubated at room temperate for 2 hours on a microplate shaker. After washing four times, 100 µL of detection buffer (HRP-conjugated anti-human IgE monoclonal antibody) was added to all wells and the plate was sealed again for 1 hour at room temperate, on a shaker. After washing four times, 100 µL of tetramethylbenzidine (TMB) substrate solution was added to all wells and incubated for 15 minutes at room temperature, in the dark. Finally, 100 µL of 1M sulphuric acid was added to stop the reaction, and the plate was read at 450 nm using a GloMax Discover Microplate Reader (Promega, US). A graph was then plotted showing the absorbance against the concentration of the standards. The unknown values of total IgE antibodies from healthy and allergic subjects were interpolated from the standard curve using a polynomial curve fit (GraphPad Prism 9.4.1 software).

**Allergen-Specific IgE ELISA**

Enzyme-linked immunosorbent assays (ELISAs) were developed in-house to measure IgE specific to the common peanut protein allergen, Ara h 2, and the lipid-binding peanut protein allergen, Ara h 8. 96-well plates (Thermo Fisher Scientific, UK) were coated with 4µg/mL of Ara h 2 (Indoor Biotech, UK) or Ara h 8 (Indoor Biotech, UK) and incubated overnight at room temperature. The plate was then washed with PBS-0.05% Tween-20 and blocked with PBS + 1% Tween-20 and 10% BSA, for 1 hour. The plate was washed again and plasma samples diluted 1:5 in assay buffer (PBS-0.05% Tween-20 and 1% BSA, purchased from Invitrogen; ThermoFisher Scientific) were added to wells and incubated for 2 hours at room temperature. The wells were washed before HRP-conjugated anti-human IgE monoclonal antibody diluted 1:100 in assay buffer was added to all wells and incubated for 1 hour at room temperate. After washing, TMB was added to wells and incubated for 15 minutes at room temperature, in the dark. Finally, 1M sulphuric acid was added, and the plate was read at 450 nm using a GloMax Discover Microplate Reader. The OD values for each sample were plotted on a graph where high absorbance indicates high allergen-specific IgE levels.

**Figure S1. Allergen specific IgE results.**

**
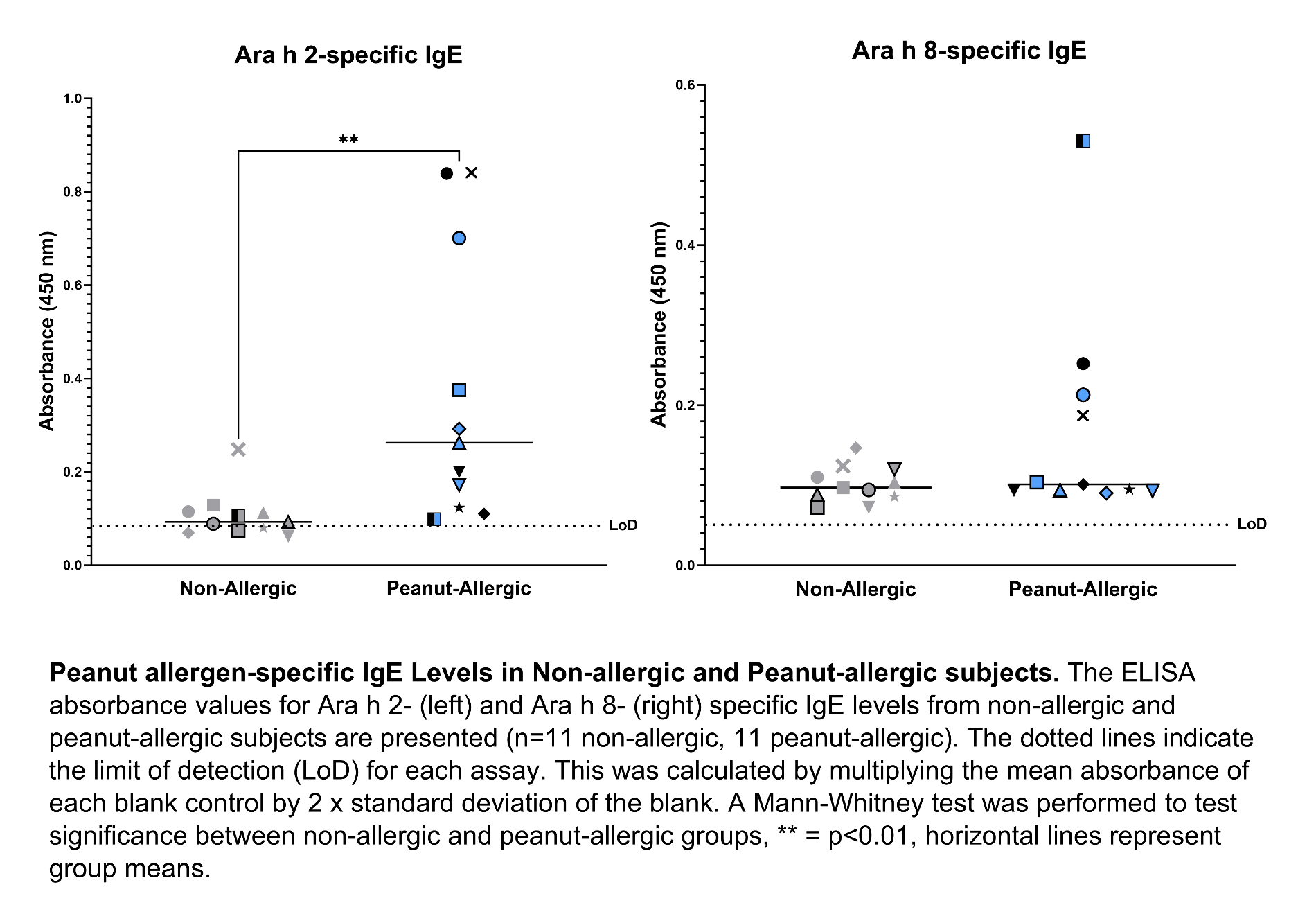
**

**Figure S2.** Specification of the peanut oil utilised in study.

**Supplementary Text 1.** Further detail on the optimisation of methods shown in Figure 2.

**Figure 2A-E** detail the optimisation of each procedure before this was applied to peanut-allergic and non-allergic samples. Increasing iNKT cell yield by was first optimised by iNKT cell expansion with the glycolipid, α-GalCer. As shown in **Figure 2Aii,** at Day 0 of culture, the iNKT cell population in this representative sample was 0.42% of lymphocytes, which expanded to 16.77% by Day 14 of culture with α-GalCer. DMSO-stimulated PBMCs showed no expansion of iNKT cells by Day 14, constituting 0.47% of CD19^-^ lymphocytes. The blank-loaded tetramer was used to detect any false positive binding of cells to the tetramer. This negative tetramer control also showed little expansion (0.80%) by day 14. These results are replicated across 9 healthy donors (**Fig. 2Aii)** which highlight iNKT cells constituted a mean of 0.69% (SD=0.13) of CD19- lymphocytes before iNKT cell expansion. After 14 days of stimulation with α-GalCer and IL-2, the percentage of iNKT cells increased to 8.20% (SD=5.02), with all donor PBMCs showing some degree of iNKT cell proliferation. A two-way ANOVA revealed this Day 14 iNKT cell population was significantly different to the DMSO control of 0.51% iNKT cells (SD=1.30, p<0.0001) and the Negative GalCer Tetramer control of 0.21% iNKT cells (SD=0.28, p<0.0001).

The isolation of iNKT cells after expansion was then successfully demonstrated using the α-GalCer-loaded CD1d PE tetramer where iNKT cells were fluorescently tagged, and subsequently isolated from the PBMC culture by immunomagnetic isolation. **Figure 2Bi** shows a representative example of iNKT cell isolation, highlighting an iNKT cell population of 16.77% before isolation. After immunomagnetic isolation, the resulting iNKT cell purity is 89.03%. The isolation of iNKT cells achieved an average purity of 83.34% (n=7, SD=7.88) (**Fig 2Bii**).

As human monocyte-derived DCs (hmoDCs) were generated, CD14+ Monocyte isolation was tested and consistently achieved high purities, averaging 93.86% (n=9, SD=3.30) after isolation (data not shown). Once monocytes were stimulated to generate iDCs, the cells were stimulated with α-GalCer and maturation markers were measured by flow cytometry. The standard method of maturing iDCs with LPS was adopted as a positive control. The relative median fluorescence intensity (rMFI) of surface makers expressed on iDCs, LPS-matured DCs, and α-GalCer-stimulated DCs are presented in **Figure 2C**. Across 4 healthy subjects, there was significant upregulation of CD80 (p<0.01), CD86 (p<0.05), CD40 (p<0.05), and HLA-DR (p<0.05) in LPS-stimulated DCs, compared to iDCs. This upregulation indicates LPS stimulation successfully matured iDCs However, paired t-tests reveal no significant upregulation of maturation markers expressed on α-GalCer-stimulated DCs (p<0.05).

Also, the ability of DCs to internalise lipids was demonstrated utilising a fluorescent derivative of α-GalCer, dansylated α-GalCer. This was utilised and imaging cytometry conducted to analyse whether the iDCs internalised the fluorescent α-GalCer. DCs were cultured with dansylated α-GalCer and flow cytometry plots shown in **Figure 2Di** indicate 50.61% of DCs had positive expression of α-GalCer and also positive for CD1d, the molecule crucial for presenting lipids. Imaging flow cytometry of this internalisation is presented in **Figure 2Dii,** where α-GalCer (purple) can be visualised inside of the DC. Using IDEAS Software (v6.2, Luminex), the Internalization feature was performed, which reports the median internalization quotient, MIQ, where cells with high cell internal fluorescence has a positive score, those with balance fluorescence have a score around zero, and those with little internal fluorescence have a negative score. The results here showed a MIQ of 2.4, showing high DC internalisation of α-GalCer. Thus, despite no effect on DC maturation, this imaging cytometry confirmed that α-GalCer had been internalised by DCs, and can be co-expressed with CD1d.

Finally, the timing of iNKT:DC co-culture was determined. **Figure 2Ei** demonstrates a significant decrease in the percentage of iNKT cells producing IFN-γ from 0-5 h to 24-29 h of co-culture with α-GalCer-stimulated DCs. This suggested 0-5 h is the optimal co-culture length to detect the rapidly produced IFN-γ. As all iNKT cells were expanded with α-GalCer before co-culture with α-GalCer-stimulated or DMSO-stimulated DCs, there was high levels of IL-4 produced before co-culture due to activation from expansion. Thus, the percentage of iNKT cells producing IL-4 was high before co-culture, and then maintained high after co-culture with α-GalCer and DMSO stimulated DCs, at both 0-5 h and 24-29 h. Hence, there were no significant differences in IL-4 production between both time points in the α-GalCer or DMSO control (p>0.05).

**Table S1. Demographics of Subject Subset used for Cytokine Analysis.** The demographics of the 6 peanut-allergic and non-allergic subjects who returned for further blood donations, and thus their data was used to perform iNKT and DC cytokine analysis. The table specifies their age, sex, ethnicity, form of peanut allergy diagnosis, any other allergies, total serum IgE levels, and Ara h 2/Ara h 8 specific IgE levels.

|  | Non-Allergic | Peanut-Allergic |
| --- | --- | --- |
| Number of Participants | 6 | 6 |
| Mean Age (Range) | 32.8 years (20-54) | 24.5 years (21-32) |
| Sex | Female: 4  Male: 2 | Female: 4  Male: 2 |
| Ethnicity | White British: 4  White Polish: 1  Latino: 1 | White British: 5  Mixed Race: 1 |
| Peanut Allergy Diagnosis | N/A | GP-confirmed: 6  Positive RAST and/or positive Skin-prick test: 6 |
| Other IgE Allergies | N/A | Cats: 4  Birch Pollen: 3  Grass Pollen: 2  Dust mites: 2  Other nuts: 2  Cucumbers: 1  Courgettes: 1  Apples: 1  Pears: 1  Peaches: 1  Cherries: 1  Dogs: 1  Mould: 1 |
| Mean Total IgE (Range) | 62.4 ng/mL (7.4 - 151.3) | 210.4 ng/mL (53.4 – 605.8) |
| Mean Peanut-specific IgE (Range) | Ara h 2  0.097 O.D. (0.062 – 0.129)  Ara h 8  0.112 O.D. (0.072 – 0.129) | Ara h 2  0.368 O.D. (0.099 – 0.839)  Ara h 8  0.214 O.D. (0.090 – 0.530) |

**Figure S3.** iNKT Cell gating strategy.


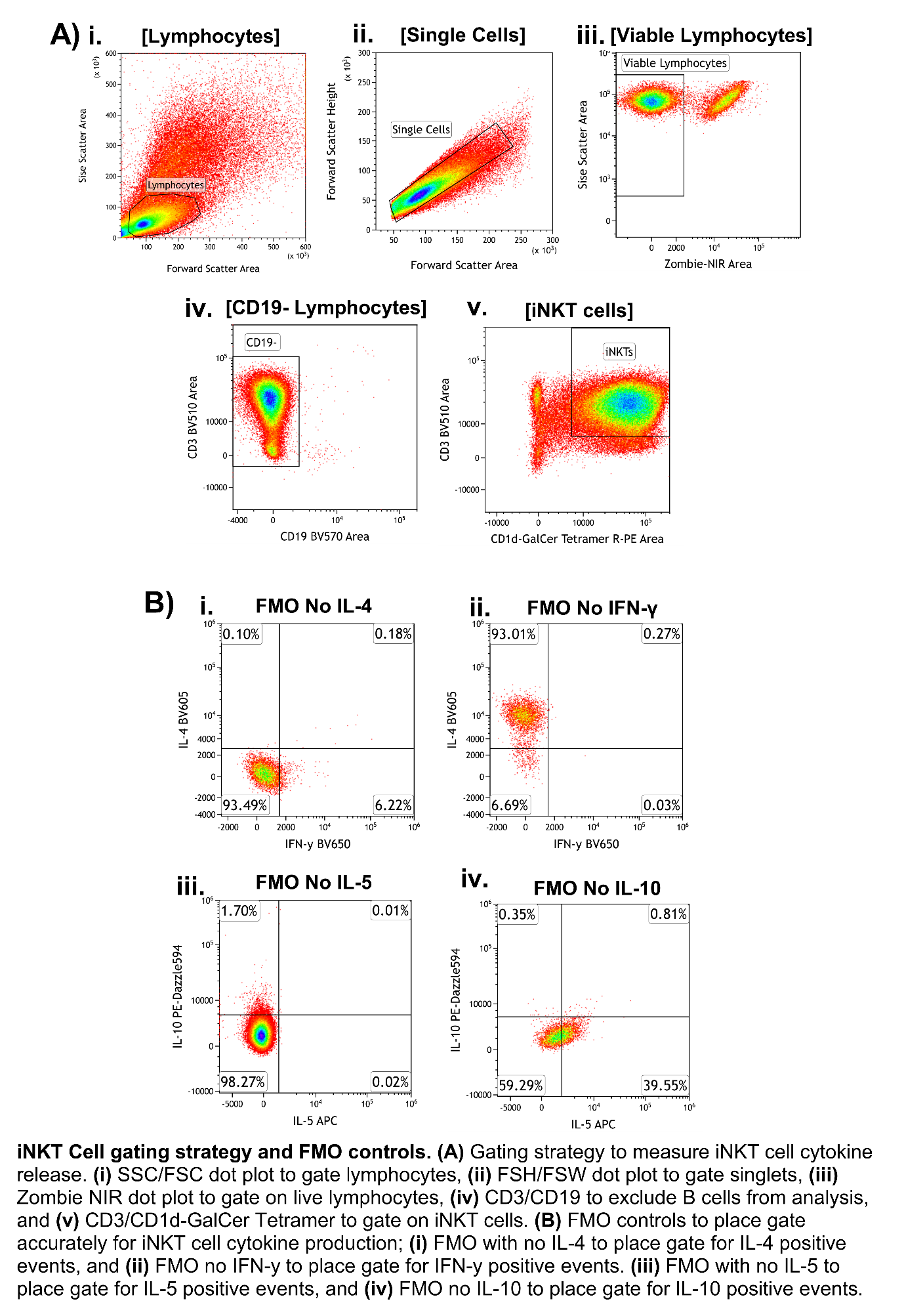


**Figure S4.** Representative FACS plots of iNKT cell cytokine production.


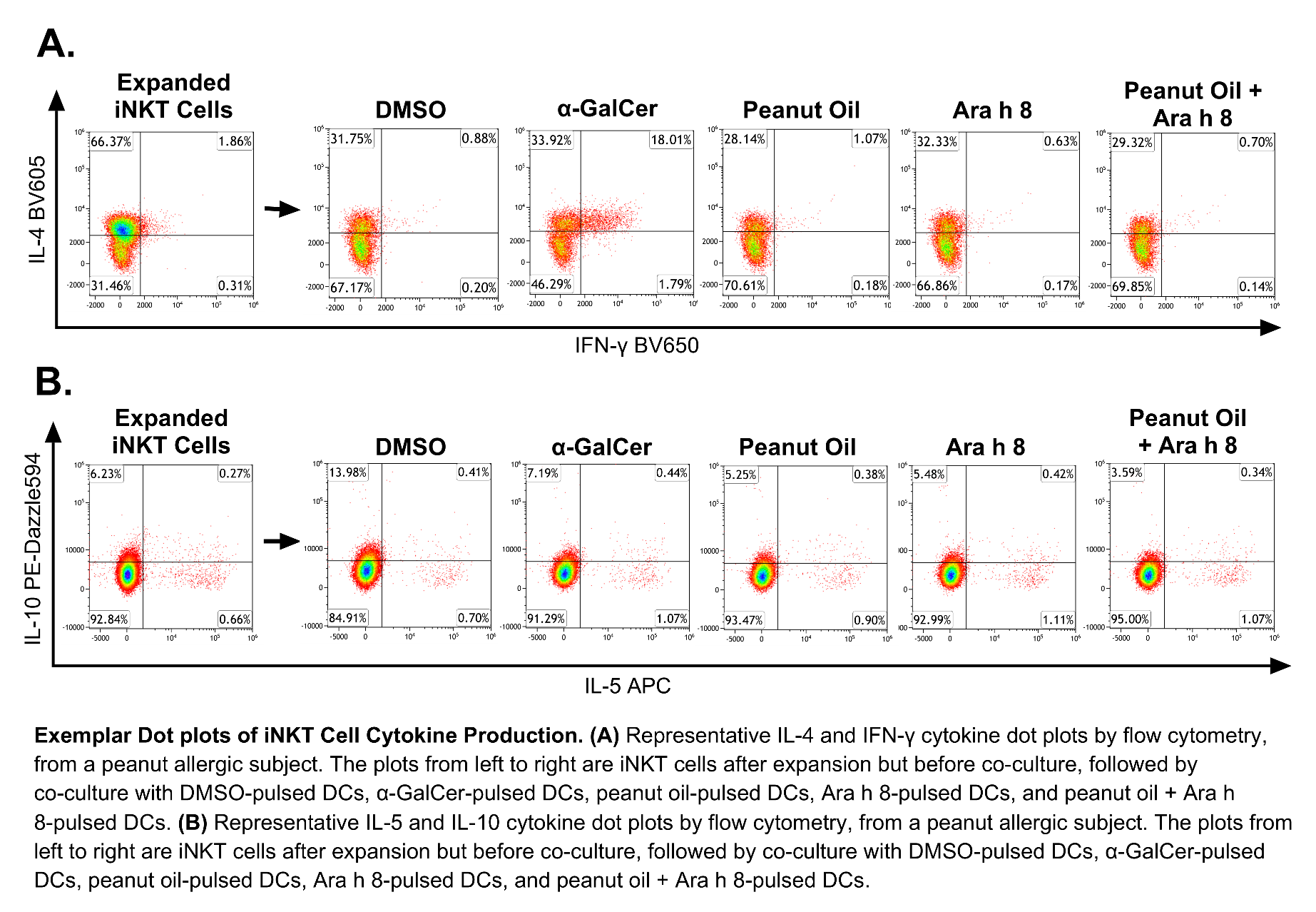


**Figure S5**. α-GalCer clustering analysis.


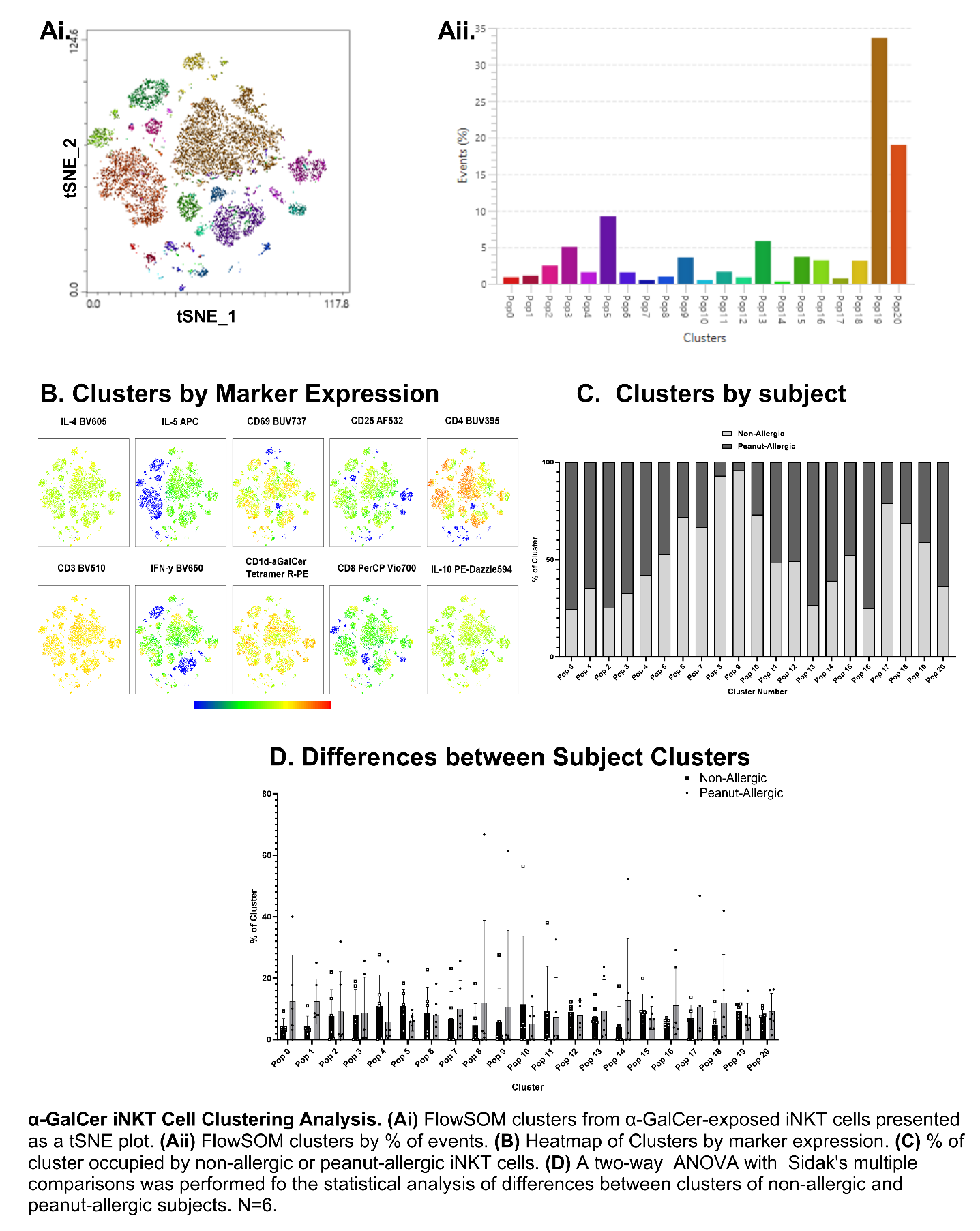


**Figure S6.** DC gating strategy.


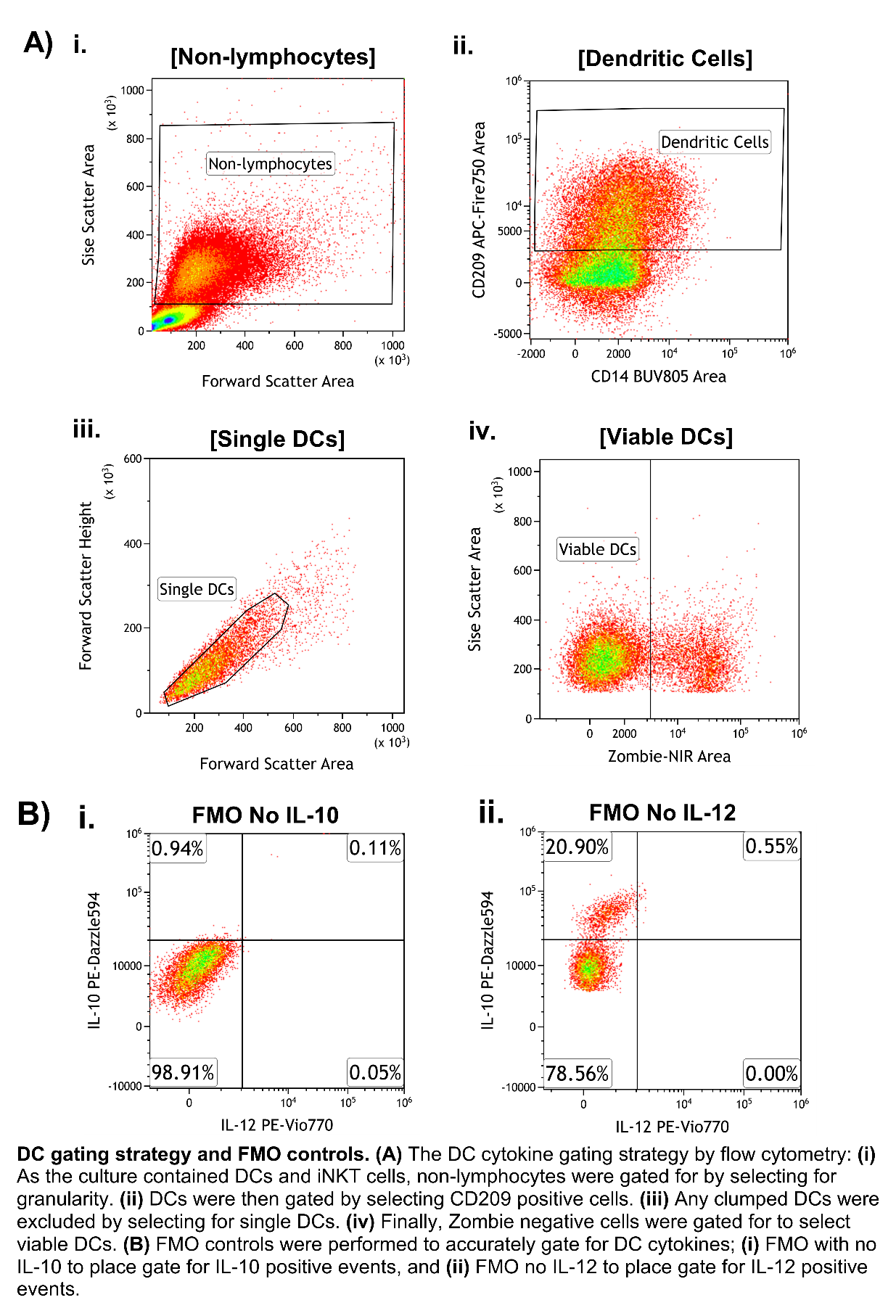


**Figure S7.** Representative FACS plots of DC cytokine production.


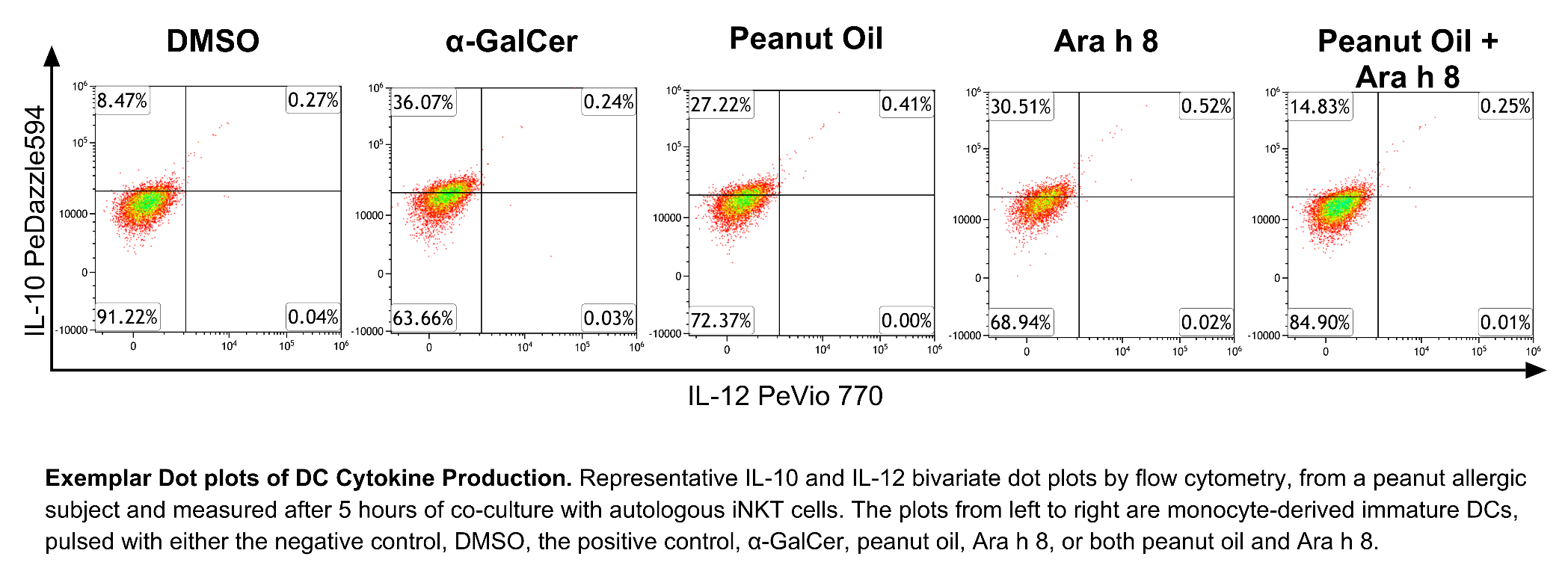

Supplement: Supplementary file 1 [file DataSheet_1.docx]
